# Electrical stimulation to clinically identify position of the lingual nerve: results of 50 subjects with reliability and correlation with MRI

**DOI:** 10.1007/s10006-021-00985-5

**Published:** 2021-07-13

**Authors:** Sanaa Aljamani, Callum Youngson, Fadi Jarad, Francis O’Neill

**Affiliations:** 1 Restorative Unit, School of Dentistry, University of Liverpool, Liverpool, UK; 2 School of Dentistry, University of Jordan, Amman, Jordan; 3 Oral Surgery Unit, School of Dentistry, University of Liverpool, Liverpool, UK

**Keywords:** Lingual nerve, Third molar surgery, MRI, Electrical stimulation, Nerve mapping

## Abstract

**Purpose:**

Recently we described mapping of the lingual nerve clinically in patients using electrical nerve stimulation. This paper reports results of a larger study with inter- and intra-observer reliability and comparison with positional measurements from magnetic resonance imaging (MRI).

**Methods:**

In 50 healthy participants, measurements were taken when subjects felt a tingling sensation in the tongue induced by a stimulation probe over the lingual nerve. Three positions were measured in relation to the third molar. Measurement reliability was tested for both inter-observer and intra-observer agreement and positional data of the lingual nerve measured clinically was also compared with nerve position as measured from MRI scans.

**Results:**

Out of 50 participants, 96 nerves (49 = left/47 = right) were included in the study. The lingual nerve was identified in 90% (87) of this sample. The mean of height of the nerve in points A, B and C were 9.64 mm, 10.77 mm and 12.34 respectively. Inter-and intra-observer agreement was considered to be good to excellent (ICC = 0.8–0.96). Agreement between nerve mapping measured values and MRI measured values was good (ICC < 0.6).

**Conclusion:**

This technique may prove useful for the clinical determination of lingual nerve position prior to procedures in the third molar region.

## Introduction

Lower third molar extraction is one of the most commonly performed surgical procedures in the oral cavity [1]. It is however associated with an incidence of nerve damage affecting either the inferior alveolar nerve or the lingual nerve [1, 2]. Several factors are related to a higher risk of nerve damage such as proximity of the nerve to the roots of wisdom teeth as well as other patient-related factors [3, 4]. Some of these factors can be studied clinically or radiographically.

Lingual nerve damage has a negative impact on quality of life due to impairment of speech, swallowing, taste and persistent pain all of which can interfere with normal activities of daily living [5, 6]. This has also raised medicolegal concerns and the profession should therefore be alert to the importance of assessment of this nerve to reduce the risk of damage [7].

Unlike the inferior alveolar nerve, whose position is identified on radiographs by the bony markings of its canal, the exact position of the lingual nerve cannot be identified in a routine pre-operative exam. Knowledge of its position clinically may be important as lingual nerve injury has been seen not just after third molar surgery but also after periodontal currettage of the distolingual of the second molar, after wedge resection of the retromolar pad and from screw placement at the angle of the mandible.

The anatomy of the lingual nerve around the third molar region has mainly been studied in cadaver dissections [8–10]. Limited literature is also present in utilising imaging techniques such as magnetic resonance imaging (MRI) and ultrasonography [11–14]. In the clinical setting, those techniques cannot be practically used for chairside examination of the lingual nerve for pre-operative assessment. Recently, we demonstrated another technique of a chairside electrical stimulation using a stimulator device which is available to most dental practitioners [15]. Out of 20 healthy participants, 18 were able to discriminate the sensation, the position was recorded and there were minimal adverse events. The aims of this study are to expand the sample size and examine both inter- and intra-observer reliability of measurements from nerve mapping, along with a comparison to positional data from magnetic resonance imaging.

### Aims and objectives

1. To test the reliability of this method by performing intra-observer and inter-observer agreement.
2. To corroborate the experimental clinical results with positional data from high-resolution MRI scans.

## Materials and methods

This study was approved by the Research Ethics sub-committee for physical interventions of the University of Liverpool and all participants signed an informed consent agreement. Healthy participants were recruited to the study in the time between September 2016–2018. Participants were excluded if they had implanted devices, e.g. pacemaker, severe gag reflex, ulceration or similar condition of the mucosa in the area studied, known neuropathy or pain in the nerve being studied, an absence of wisdom tooth and or lower molars (Extracted or known as developmental missing), inability to consent or below the age of 18 years.

**Fig. 1:**
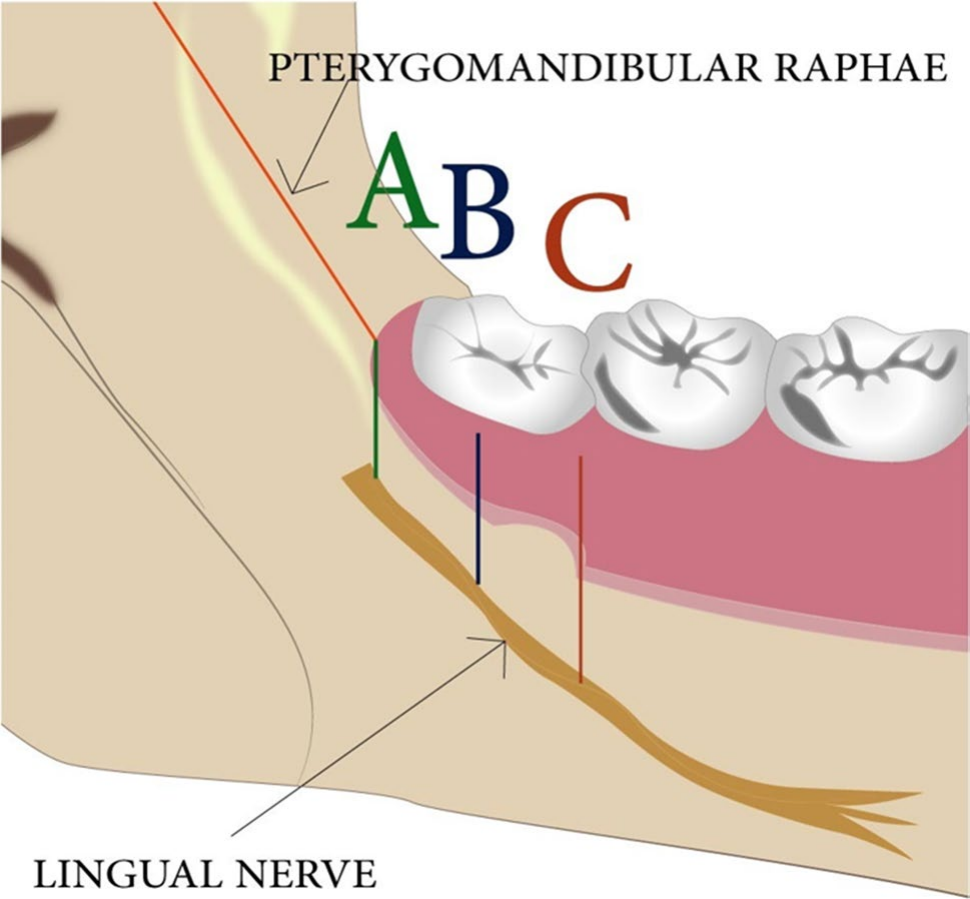
Showing a section of the mandible in anterior–posterior position. This views the mandibular lingual side and the relation of the anatomic position of the lingual nerve in regard to the reference points: A, B and C

Lingual nerve stimulation was performed in both right and left side whenever possible. This technique been described in full previously [15]. A brief description follows:

An electric stimulating device was used to provide electrical impulse to the lingual nerve (Gentle Pulse™, Parkell Edgewood, NY, USA). This device has a fixed current, variable voltage (− 150v to + 450v), externally controllable output. A semi-sawtooth current waveform with 300-μs pulse width produced in bursts of 7 pulses separated by 15 ms at a frequency of 5 Hz per burst. A tingling or vibrating sensation on the lateral aspect of the tongue reported by the subject indicates stimulation of the lingual nerve. The nerve was identified in three different areas, points A, B and C (see Fig. 1). This was performed in relation to clinical and anatomical landmarks intra-orally. Point A refers to the retromolar pad area which lies at, or in front of, the pterygomandibular raphe attachment. Point C referred to the distolingual attached gingivae of the erupted lower second molar. Point B referred to the midpoint of the attached lingual gingivae of the third molar crown. In the case of complete eruption, this becomes easy to identify, whereas in full, or partial, impaction, this point had to be estimated as the midway point between point A and point C. Measurements were taken from the middle of the electrode position to the gingival crest at the second molar and in erupted third molars or the bottom of the retromolar pad in unerupted third molars as described previously [15].

### Inter- and intra-observer reliability testing

Ten percent of the overall sample size for both intra-observer and inter-observer reliability testing was included in this part of the study. This was based on the number of the lingual nerves rather than number of participants. At least 10 lingual nerves were mapped by two independent observers. For inter-observer ratings, whenever possible, a recruited participant had both their right and left lingual nerves mapped and, following the first set of measurements, an additional set of measurements were taken by a different operator with at least 2 weeks between different observer’s measurements. For intra-observer ratings, the same subject was re-tested by the same operator with at least 2 weeks between observation measurements.

### MRI imaging corroboration

In order to corroborate nerve position data gained from the nerve mapping part of the study, positional data from MRI scans was acquired from 10% of lingual nerves studied. This data was acquired on the 3 Tesla MRI (Siemens Magnetom Prisma). Participants were selected randomly and were screened to ensure there were no contra-indications for MRI and claustrophobic patients were excluded.

**Fig. 2:**
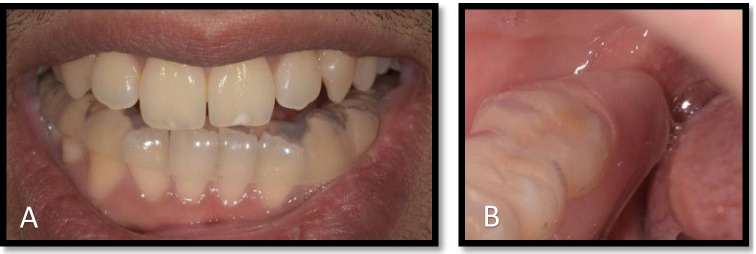
Showing intra-oral tongue separator (**A**) to create an air space between the tongue and the lingual mucosa (**B**)

**Fig. 3:**
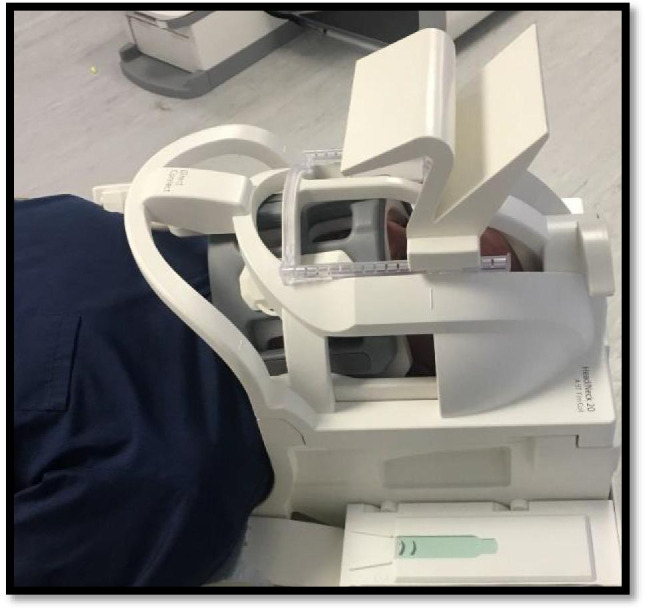
Showing extra-oral coil configuration and participant positioning used to optimise the signal around the field of view

The pre-scanning set up used a custom made intra-oral splint that extended to the lingual side of the posterior mandible to create a space between the lingual tissue of the mandible and the tongue (see Fig. 2) to increase the physical separation between these tissues. This allowed us to clearly demarcate the border of the tongue and lingual mucosa covering the mandible thereby clarifying the anatomical boundaries. In order to enhance image quality, the scanning protocol was developed utilising extra-oral head coils (see Fig. 3) and 3D-double echo steady state with water excitation (3D DESS WE). This has been reported to give a better definition of extra-cranial nerve branches [12, 16].

The final scans acquired different sequences comprised as follows:

1. Coronal T1-weighted STIR sequence of 2-mm slice thickness.
2. Coronal T2-weighted DE 3D WE sequence of 0.6-mm slice thickness.
3. Axial T2- weighted DE 3D WE sequence of 0.7-mm slice thickness.
4. Axial T2-weighted DE 3D WE sequence of 0.6-mm slice thickness.

The MRI scans were viewed using SyngoVia software on a 24-inch workstation. Two observers were involved in viewing the lingual nerve, a postgraduate student investigator and a consultant oral surgeon. Observations of MRI measurements were made on images that were anonymised after acquisition in order to blind observers. The lingual nerve was located as it exited from the base of the skull at the foramen ovale and its course followed until it got to the lingual surface of the mandible, adjacent to the third molar. This was done alternating between axial view and coronal views. Coronal views of 0.6-mm slice thickness 3D DESS WE were used for taking measurements of the lingual nerve. A standardised data collection protocol was used: (1) Define the distal of the second molar tooth as the initial landmark. (2) Measurement of point C reading from top of the distal interproximal gingival soft tissue. (3) In erupted third molars, point B was taken as the midpoint of the crown the tooth. In unerupted molars, it was taken as the midpoint between points A and C. (4) Point C was derived from the clinical measurement between A and C taken during nerve mapping. This distance in mm was divided by 0.6 (slice thickness) to give number of slices movement from point C to arrive at point A. Any uncertainty over identification of particular areas of hyperdensity can be resolved by comparing these with the course of the nerve followed and in addition comparing anatomical structures with the T1-weighted images also generated to exclude position of vessels, ducts etc.

### Statistical analysis

#### Nerve mapping

Simple descriptive statistics were performed to analyse the clinical data from nerve mapping. Data evaluation was performed using statistical analysis software (SPSS® IBM Corporation Statistics).

The use of intra-class correlation coefficient was utilised to test (a) the reliability in inter- and intra-observer agreement and (b) Reproducibility and agreement between the electrical stimulation of the lingual nerve and MRI scans in identifying the lingual nerve height. Amongst different forms of ICC, two-way mixed ICC was chosen. This was justified for the following reasons: (1) The test is concerned in measuring the agreement between these two methods, or observers only, irrespective of other methods or observers. (2) These two methods, observers are measuring the same participants using the same reference points to get a continuous measurement. (3) The absolute agreement is performed to identify the extent to which each measurement taken from the MRI corresponded to measurements from nerve mapping [17, 18].

## Results

### Demographics

**Fig. 4:**
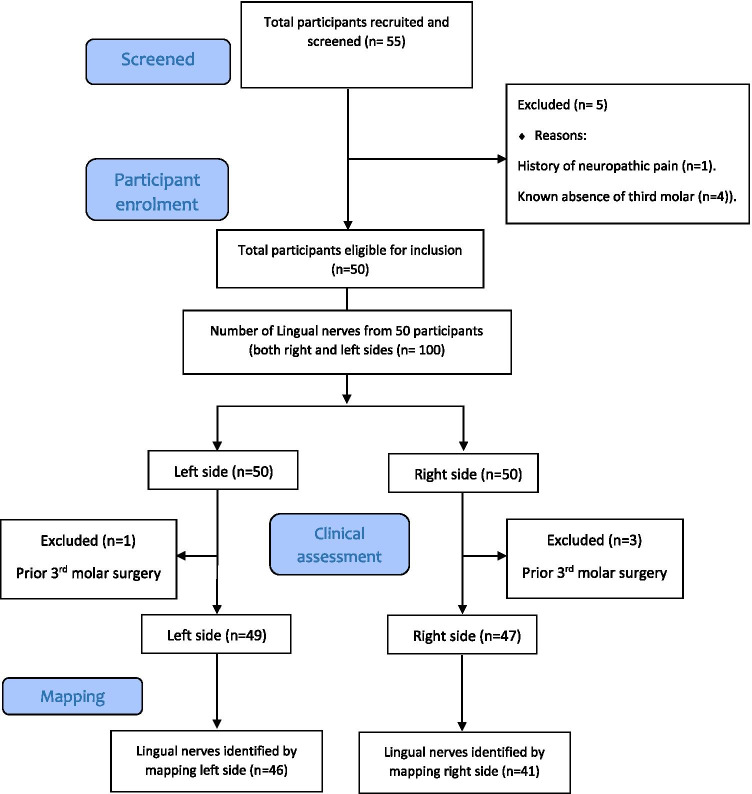
Participant flow chart

In total, 55 participants were recruited initially. Five participants did not meet inclusion criteria. A total of 50 healthy participants were included (22 males and 28 females). The ages ranged from 18 to 38 years of age and the mean age was 24.1 years. The ethnic origin of the study participants consisted of 60% white British and 40% of other races including African, Arabic and Asian (see Table 1 for details). Of these 50 participants, 3 had had extraction of the third molar on the right side and 1 on the left side so the lingual nerve on those sides was not included but the remaining side was. Therefore, all together 50 participants were included with 47 lingual nerves on the right side included and 49 lingual nerves on the left side, see study flow chart Fig. 4.

**Table 1:** Showing the detailed demographic data of the participants in the clinical mapping study

Demographic data	Distribution
Gender	
Female	28
Male	22
Age range	
18–28	36
28–38	14
Ethnic origin	
White British	28
Asian	17
Arabic	2
African	3
Total	50
Status of the third molar in both right and left side of each participant	
Erupted	37
Partially erupted	29
Unerupted	30

The status of the third molar amongst the sample is relatively homogenous in the studied sample

Of the overall 96 lingual nerves that were included in the study, 87 lingual nerves were able to be mapped using the electrical stimulation 41 on the right side and 46 on the left side.

### Lingual nerve position

**Fig. 5:**
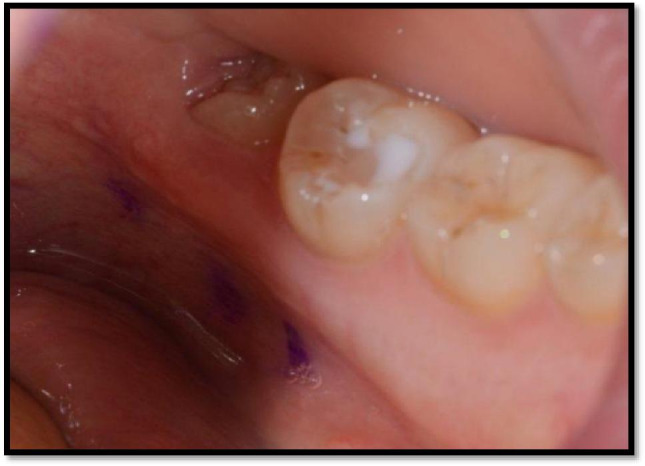
Illustrating an intra-oral photograph showing a left side mapped lingual nerve in a partially erupted third molar region. Notice points A, B and C with a relatively intermediate height (between 5 and 10 mm)

Positional data of lingual nerve mapping was collected from the three identified landmarks A, B and C. This was attempted in all 50 participants. The mean height of the lingual nerve at point A was 9.64 mm (SD 2.98), point B 10.77 (SD 2.76) and point C was 12.34 (SD 3.16). Detailed descriptive results are shown in Table 2. An example of the visualisation of the points measured is shown in Fig. 5. Although there was a trend towards smaller mean height measurements for the erupted third molars in most of the measured points, this was not significantly different between partially erupted and unerupted molars over the range of data points. Also, as the age range in this particular population was quite narrow (18–38 years), there was no significant difference in mean nerve height seen between age groups.

**Table 2:** Showing the frequency of valid and missed data for each point and the mean value with standard deviation for each point

Points of the mapped lingual nerves clinically	Point A	Point B	Point C
Total identified	73	75	79
Total unidentified	23	21	17
Total sample	96	96	96
Mean value of the height (mm)	9.64 mm	10.77 mm	12.34 mm
Standard deviation total (mm)	2.98 mm	2.76 mm	3.16 mm
Points less or equal to 5 mm	5	2	1

Point C was the most identified point, and this could be due to anterior position of this point which facilitates identifying it amongst the rest. The low standard deviation of the measurements indicates the close proximity of the overall data to the mean value

### Adverse events

Throughout the study, the participants were able to report on any adverse events after applying the electrical stimulus on the soft tissue in the lingual area and these were described previously [15] and included (a) bad taste from the ink, (b) inadvertent dental pulp stimulation, (c) twitch sensation of the floor of the mouth, (d) pins and needles and (e) elicited gag reflex (Table 3). These were similar for the larger group in this study. One participant reported temporary paraesthesia, which he described as a mild tingling of the tongue during eating. This completely resolved within 3 days. Table 3 shows the detailed feedback on any lasting sensation and its duration following each application.

**Table 3:** Showing the lasted sensation after applying the electrical stimulus

Reported lasted sensations following stimulation (i.e. tingling, vibrations, numbness or pins and needles)	Number of participants
No identified lasting sensations	38
Lasting sensation less than 1 min	8
Lasting sensation more than 1 min (up to 7 min)	3
Lasting sensation up to 3 days	1
Total number of participants	50

The majority reported no lasting sensation. Only one had a tingling sensation that lasted up to 3 days

### Agreement studies

**Fig. 6:**
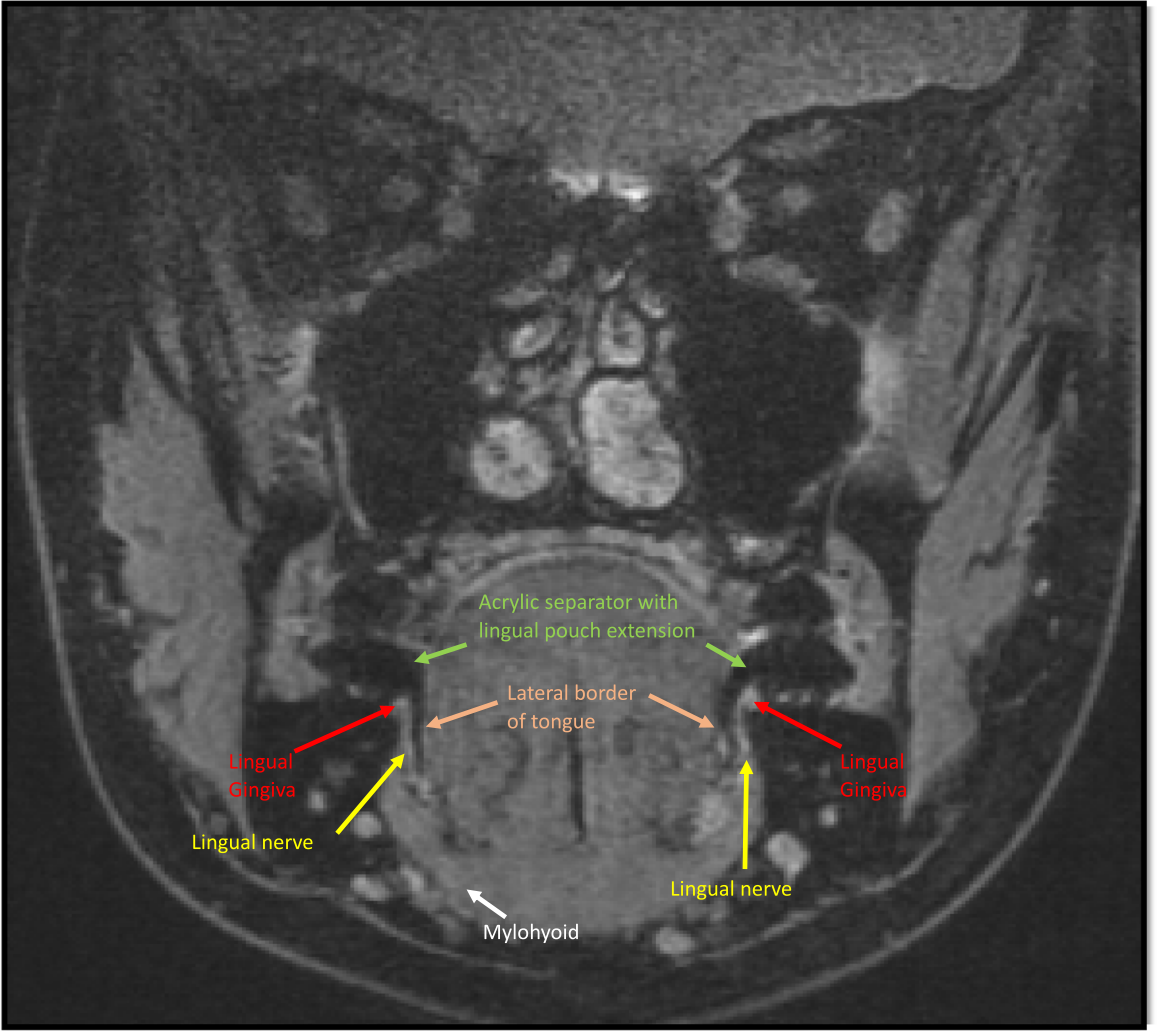
Annotated MRI coronal view of T2 3D-DESS WE at the level of an erupted third molar region, (point B) notice the intra-oral separator in black giving clear demarcation of the tongue and lingual mucosa of the mandible. Structures identified—lingual nerve (yellow arrow), lingual gingiva (red arrow), lateral border of tongue (pink arrow) and mylohyoid muscle (white arrow)

Agreement between different observers (inter-observer), different trials same observer (intra-observer) and different techniques (MRI vs nerve mapping) in identifying A, B and C was performed on 10% of the original sample size (10 lingual nerves). These demonstrated good to excellent agreement. Inter-class correlation between observers was 0.80–0.96 depending on position and between 0.82 and 0.95 for intra-observer observations. Agreement between values obtained by lingual nerve mapping and those obtained via measurements from MRI scan images was moderate to good falling between 0.68 and 0.92 depending on position. Demographics of MRI participants are shown in Table 4 and detailed results with 95% confidence intervals are shown in Table 5. An example of the readings obtained from measurements taken from the MRI images is shown in Fig. 6 and Table 6.

**Table 4:** Showing the demographic data of the MRI study

Demographic data	Number (percentage)
Males	2 (40%)
Females	3 (60%)
Total participants	5 (100%)
Age range	21 to 30 y
Mean	25y
Total nerve scanned	10
Total identified nerves	10
Status of the third molar in both right and left side of each participant	
Erupted	2
Partially erupted	1
Unerupted	7

**Table 5:** Detailed descriptive and ICC results of all study parts

Point/test used	Inter-observer agreement ICC and (95% CI)	Intra-observer agreement ICC and (95% CI)	MRI vs nerve mapping agreement (95% CI)
Point A	0.96 (0.8–0.9)	0.82 (0.4–0.9)	0.92 (0.5–0.9)
Point B	0.80 (0.4–0.9)	0.95 (0.8–0.9)	0.82 (0.3–0.9)
Point C	0.93 (0.7–0.9)	0.95 (0.8–0.9)	0.68 (0.06–0.9)

Note that ICC results across the points show good to excellent agreement with relatively narrow range of confidence interval. This relatively indicates more accurate results. It is worth mentioning that identifying point C between nerve mapping and MRI showed the least agreement with wide CI. This can be justified by the complexity of oral structure around that point that impaired the visualisation by MRI

**Table 6:** Showing the demographics of participants in the MRI correlation with clinical nerve mapping

Descriptive details	MRI
Participants (male:female)	5 (3:2)
Total nerves scanned	10
Total nerves identified	10
Identified points A	8
Identified points B	8
Identified points C	9

## Discussion

This study was designed to investigate the use of electrical stimulation to identify the in situ position of the lingual nerve. The technique was able to identify the vertical position of the nerve. The mean heights of the mapped lingual nerves in lingual soft tissue at points A, B and C were 9.6, 10.7 and 12.3 mm respectively. These measurements lie within the reported heights in the literature (2.06 to 16.8 mm) [4, 8–11, 19–21]. The anatomical landmarks used in the current study are within the overlying soft tissues compared to bony landmarks of the alveolar crest in most of the prior literature which may affect their direct comparison.

5.7% of the total 87 lingual nerves identified in this study were located in the region of the alveolar crest. This was a smaller percentage than most reports in the literature such as Miloro of 10% [11], Behnia of 14% [10] and Benninger of 14% [14], but close to Kieselbach’s observation of 4.7% [8].

Whilst inter- and intra-observer reliability showed good and excellent agreements respectively, some of the repeated measurements could not be detected, and therefore, their paired points of the sample were not able to be allocated. Calculating the agreement in these cases was performed by removing those paired points from the calculation as there were no numerical value of the unidentified point. This reduced the number of pairs included.

In the comparison of our mapping data with positional data from magnetic resonance imaging, the interclass correlation coefficient showed good to excellent level of agreement between the two different methods. The available literature imaging the course of the lingual nerve is relatively limited. Some authors study the lingual nerve in an anatomically more superior position near the base of the skull [12] and others have explored the lingual nerve in the third molar region but in cases where the normal anatomy has been distorted due to nerve injury in the detection of neuropathy and neuroma formation (Cox et al. 2016).

Miloro and colleagues are one of the few groups to have looked at the lingual nerve in the third molar region in normal subjects without prior nerve injury [11]. They studied the mean vertical and horizontal positions of the lingual nerve in relation to the alveolar crest and they found the mean height in 20 lingual nerves was 2.75 mm (range 1.52–4.61 mm). Their study, however, used the bony alveolar crest as the anatomical reference point for measurements. This is almost 2.5–3 mm deeper than the overlying soft tissue reference point in the study we present here, which correlates well with the depth of the crevicular sulcus and the biological width. In order to compare our own results with those of Miloro’s previous findings, compensation for the biological width and thickness of the overlying mucosa needs to be taken into account [22]. Added to that, the normal width of the lingual nerve being 1.5–2.5 mm and the potential maximum depth at which a nerve could be stimulated whilst the superior portion of the nerve is at the level of the alveolar crest becomes 5 mm. Therefore, to compare our results with those of Miloro, we must subtract 5 mm from our presented measurements. This has been explained more fully previously [15].

In the inter-observer reliability testing experiment, several points of the nerve mapping were not able to be identified by both observers, although could be identified by one observer. This most likely was due to issues with moisture control where, unless the field was dry, the subject did not feel the stimulation. This could be improved with the addition of a tongue aspirator which was not available at the time. Measurements could also be improved with a measuring tool such as a calliper to reduce the chance of the angulation of the observers eyeline affecting the reading of measurements off the ruler.

Visualising the lingual nerve with magnetic resonance imaging in this study was challenging; this was facilitated by using both intra-oral (tongue separator) and extra-oral measures (coil arrangement) and also by following the course of the nerve from the foramen ovale to its periphery to enhance identification of the nerve. Future studies may benefit from use of higher strength 7 Tesla MRI.

In this study, we have demonstrated that electrical stimulation of the lingual nerve with a proprietary electrical stimulation device is feasible, safe and practically achievable. Results from this technique are similar to those recorded in prior literature from. The technique showed good reliability between intra- and inter-observer agreements and has potential for further development in preoperative screening of lingual nerve position.
